# Supplementary material for: Content-rich biological network constructed by mining PubMed abstracts
Source: BMC Bioinformatics. 2004 Oct 8;5:147. doi: 10.1186/1471-2105-5-147 (PMC528731; doi:10.1186/1471-2105-5-147)
Supplement: Additional File 5 — The original Chilibot query results of the term "long-term potentiation (LTP)" and 22 other terms, limiting the latest references analyzed to the years 1990, 1995, 2000, and 2004. [file 1471-2105-5-147-S5.bz2 › chilibotAdditionalFile5/ltp2004/html/TRKB_ARC.html]

 


 **TRKB** and **ARC** 
  
Found 2 abstracts in PubMed,  **2 abstracts were retrieved and analyzed**.  


---

 Search Google  |
 PDF files only 
|  EDU domain only 

---

- Proc Natl Acad Sci U S A, 2002   **The brain derived neurotrophic factor enhances synthesis of **Arc** in synaptoneurosomes.** .
  Protein synthesis in neurons is essential for the consolidation of memory and for the stabilization of activity dependent forms of synaptic plasticity such as long term potentiation LTP .
  Activity dependent translation of dendritically localized mRNAs has been proposed to be a critical source of new proteins necessary for synaptic change.
  mRNA for the activity regulated cytoskeletal protein, **Arc**, is transcribed during LTP and learning, and disruption of its translation gives rise to deficits in both.
  We have found that selective translation of **Arc** in a synaptoneurosomal preparation is induced by the brain derived neurotrophic factor, a neurotrophin that is released during high frequency stimulation patterns used to elicit LTP.
  This effect involves signaling through the **TrkB** receptor and is blocked by the N methyl d aspartate type glutamate receptor antagonist, MK801.
  The results suggest there is a synergy between neurotrophic and ionotropic mechanisms that may influence the specificity and duration of changes in synaptic efficacy at glutamatergic synapses.
